# Age-Dependent Changes in the Proteome Following Complete Spinal Cord Transection in a Postnatal South American Opossum (*Monodelphis domestica*)

**DOI:** 10.1371/journal.pone.0027465

**Published:** 2011-11-16

**Authors:** Natassya M. Noor, David L. Steer, Benjamin J. Wheaton, C. Joakim Ek, Jessie S. Truettner, W. Dalton Dietrich, Katarzyna M. Dziegielewska, Samantha J. Richardson, A. Ian Smith, John L. VandeBerg, Norman R. Saunders

**Affiliations:** 1 Department of Pharmacology, the University of Melbourne, Parkville, Victoria, Australia; 2 Department of Biochemistry & Molecular Biology, Monash University, Clayton, Victoria, Australia; 3 The Miami Project to Cure Paralysis, University of Miami, Miller School of Medicine, Miami, Florida, United States of America; 4 School of Medical Sciences and Health Innovations Research Institute, RMIT University, Bundoora, Victoria, Australia; 5 Texas Biomedical Research Institute, San Antonio, Texas, United States of America; University of Western Ontario, Canada

## Abstract

Recovery from severe spinal injury in adults is limited, compared to immature animals who demonstrate some capacity for repair. Using laboratory opossums (*Monodelphis domestica*), the aim was to compare proteomic responses to injury at two ages: one when there is axonal growth across the lesion and substantial behavioural recovery and one when no axonal growth occurs. Anaesthetized pups at postnatal day (P) 7 or P28 were subjected to complete transection of the spinal cord at thoracic level T10. Cords were collected 1 or 7 days after injury and from age-matched controls. Proteins were separated based on isoelectric point and subunit molecular weight; those whose expression levels changed following injury were identified by densitometry and analysed by mass spectrometry. Fifty-six unique proteins were identified as differentially regulated in response to spinal transection at both ages combined. More than 50% were cytoplasmic and 70% belonged to families of proteins with characteristic binding properties. Proteins were assigned to groups by biological function including regulation (40%), metabolism (26%), inflammation (19%) and structure (15%). More changes were detected at one than seven days after injury at both ages. Seven identified proteins: 14-3-3 epsilon, 14-3-3 gamma, cofilin, alpha enolase, heart fatty acid binding protein (FABP3), brain fatty acid binding protein (FABP7) and ubiquitin demonstrated age-related differential expression and were analysed by qRT-PCR. Changes in mRNA levels for FABP3 at P7+1day and ubiquitin at P28+1day were statistically significant. Immunocytochemical staining showed differences in ubiquitin localization in younger compared to older cords and an increase in oligodendrocyte and neuroglia immunostaining following injury at P28. Western blot analysis supported proteomic results for ubiquitin and 14-3-3 proteins. Data obtained at the two ages demonstrated changes in response to injury, compared to controls, that were different for different functional protein classes. Some may provide targets for novel drug or gene therapies.

## Introduction

Several mammalian organ systems, such as the heart [1] and the central nervous system (CNS) have an increased capacity to regenerate following injury when immature compared to adult. In the CNS this has been most clearly shown in marsupial species reflecting the immaturity of their brain and spinal cord at birth compared to eutherian mammals such as rodents [2]–[3]. Thus, if the spinal cord in an opossum *Monodelphis domestica* [3]–[4] or *Didelphis virginiana* [5]–[6] is transected in the mid thoracic region in the first week of life, many axons regenerate, reaching in *Monodelphis domestica* nearly 50% [7]. At the same time a substantial growth of new undamaged axons occurs as part of normal development. These animals demonstrate near normal locomotor ability when adult [4]. However, following transection at about one month of age, growth of axons across the lesion site cannot be detected [6] and the animals have substantially impaired locomotion [8].

The inability of the adult spinal cord to regrow and repair following injury has been studied extensively for the past 30 years, particularly since Aquayo and colleagues implanted a peripheral nerve (sciatic nerve) into injured CNS tissue and showed that injured axons could grow for long distances through the graft [9]–[10]. Since then, many other types of implants have been tried and in recent years the experiments have mainly focussed on the use of stem cells (e.g. [11]–[12]). However, an important limitation of virtually all implants tried so far is that although there may be substantial axonal growth across the implant, there is very little growth outside its boundaries. The proposition is that there are many inhibitory molecular and cellular components in the adult spinal cord that prevent regeneration of injured neurites [13]–[15]. It also seems likely that complex changes in gene and protein expression as well as cellular interactions that are taking place in the immature spinal cord change during development so that the tissue goes from a state when regenerative and normal axon growth is possible to a state when it is not. Preliminary indications that numerous genes are activated in response to injury come from the studies of Nicholls and colleagues using an *in vitro* preparation of a *Monodelphis* neonatal spinal cord [16]–[18] and from our own studies in this species using mouse cDNA arrays (Super Array, SABiosystems, [19]). The advantage of a marsupial species lies in the accessibility of their newborn, which makes them amenable to *in vivo* studies. A serious limitation however, until recently has been the lack of information on gene and protein sequences in this species. The situation has been transformed by the publication of the genome sequence of *Monodelphis domestica* [20]. Unfortunately, however, there are no microarrays available for the opossum and the homology to existing microarrays is limited. We have therefore taken a proteomic screening approach to identify and define a wide range of proteins that may be involved in the response to spinal cord injury at different developmental ages. We have examined the segment of spinal cord caudal to the site of injury, since this is the region through which axonal connections have to re-grow to repair and restore effective function. In addition, this region of the spinal cord, together with the site of the lesion itself has been most studied with respect to promoting axonal growth in injured spinal cord [21]. We have compared the proteomic responses to injury at two different ages in *Monodelphis*: P7 when substantial axon growth occurs [7], [19] and P28 when it does not [8]. Additional methods such as qRT-PCR, western blotting and immunocytochemistry were performed on some proteins identified as differentially regulated after spinal injury to complement results obtained from the proteomic analyses. This study sheds light on the large number of proteins that show regulatory changes following spinal cord injury (SCI) and demonstrates that many of these changes are different at the two ages studied.

## Materials and Methods

### Animals used

#### Ethics Statement

Monodelphis domestica were obtained either from a colony based at the University of Melbourne Medical Sciences Animal House Facility, Melbourne, Australia, or from the colony at the Texas Biomedical Research Institute, San Antonio, Texas. Procedures in Australia were performed according to National Health and Medical Research Council guidelines, with the approval of the University of Melbourne Animal Ethics Committee (Ethics #0707108). Procedures in the USA were performed with the approval of Texas Biomedical Research Institute's Animal Care and Use Committee, according to the Public Health Safety Policy on the Humane Care and Use of Laboratory Animals.

Pups of both sexes were used. Day of birth was designated as postnatal day zero, P0. Animals older than 3 months were considered adult [3].

The pups were assigned to two age groups and complete spinal transections were performed at either P7 or P28. At P7 whole litters (6–7 pups) were operated on. Separate litters of pups were kept as controls as there is no consistent way to mark these very young animals without increasing the risk of cannibalisation by the mother [4]. Injuries at P28 were usually made on half the pups in a litter, since at P28 their ears can be marked. The remaining pups from these litters were anaesthetised but remained uninjured and were used as controls. Experimental and control pups were collected at 1 day (+1 d) or 7 days (+7 d) post injury. Numbers of pups used in this study are summarized in Table 1.

**Table 1:** Numbers of spinal cords collected for each age group for use in proteomics, immunocytochemistry and Western blot analyses.

Age group	Proteomics	Immunocytochemistry	Western blotting
**P7+1d**	18	4	10
**P8**	18	4	10
**P7+7d**	14	4	7
**P14**	15	3	7
**P28+1d**	8	3	6
**P29**	8	4	6
**P28+7d**	4	4	4
**P35**	4	4	4

+1d or +7d refers to days post-injury performed at P7 or P28. P8, P14, P29 and P35 are control groups.

### Spinal cord transection

At P7, *Monodelphis* pups are still attached to the mothers' teats [3]. The female adult *Monodelphis* were anaesthetized with 2–3% isofluorane; the same anaesthetic was administered to the P7 pups via a small facemask during the surgical procedure. Pups at P28 are no longer attached to the mother and were separately anaesthetized with isofluorane throughout the surgical procedure [7], [19].

Complete spinal cord transection was performed at thoracic level 10 (T10) using sharp sterilized fine scissors. Skin was closed using surgical grade glue (Vetbond, 3M, St. Paul, MN, USA). Animals were returned to their cages and allowed to recover for either 24 hours (+1 d) or 7 days (+7 d) post injury. At the end of the experimental period, control and injured animals were terminally anaesthetized with an overdose of isofluorane and spinal cords were dissected out.

Spinal cords were removed and separated into two segments, the upper (rostral to the injury) and lower (caudal to the injury) divided through the site of the injury at T10, or corresponding segments from control animal spinal cords. Spinal cord tissue was stored at −80°C until used. Only the caudal segments of the cords were used in the present study.

### Preparation of protein samples for proteomic analysis

Segments of lower spinal cords (including part of the injury area) were collected and pooled from several pups to obtain a total weight between 30 and 80 mg (Table 2) per sample. Pooled cords were homogenized 1∶10 w/v in homogenization buffer containing 0.32 M sucrose, 25 mM Tris, 1 mM MgCl_2_, pH 7 by passing tissue and buffer through 20 Gauge (G), 21G, 25G and 27G needles until no resistance was felt. Samples were centrifuged at 2000×g for 2 minutes at 4°C. Supernatant was collected for further analysis. Total protein concentration was measured using the Bradford Assay [22] with a protein standard (Sigma-Aldrich, St Louis, MO, USA) to ensure that the extraction process was comparably efficient as all samples were normalized weight to volume so the same volume could be used throughout the study.

**Table 2:** Number of cords, number of litters, pooled tissue weight and total protein concentration of samples obtained for each group of animals used in the proteomic study.

	SCI	Control	SCI	Control	SCI	Control	SCI	Control
**Age**	P7+1d	P8	P7+7d	P14	P28+1d	P29	P28+7d	P35
**Number of cords pooled**	18	18	14	15	8	8	4	4
**Number of litters used**	5	5	3	4	2	2	3	2
**Total tissue weight (mg)**	31.8	50.7	57.8	57.8	80.5	86.9	61.2	72.4
**Total protein concentration (ìg/ìl)**	26.76	18.69	15.51	13.78	18.69	21.27	16.02	15.44

+1d and +7d refer to days after injury at either P7 or P28. P8, P14, p29 and P35 are age-matched controls.

#### Sample clean-up

Aliquots of 50 µl of each sample were transferred into microcentrifuge tubes and contaminants were removed using the 2-dimensional (2D) clean-up kit (GE Healthcare Bio-Sciences Corp., Piscataway, NJ, USA) as detailed in Manufacturer's Protocol (Procedure B). Samples were centrifuged for 10 minutes at 8000×g and wash buffer was decanted without disturbing the pellet. Acetone in the wash buffer was fully evaporated before proceeding to the next step. For each age group, 6 clean-up samples were prepared.

#### Off-gel Fractionator

An Off-gel Fractionator 3100 (Agilent Technologies, Santa Clara, CA, USA) was used in accordance with the Manufacturer's Protocol. Twelve-centimetre immobilized pH gradient (IPG) strips (pH 3–10, Linear, Agilent Technologies, Santa Clara, CA, USA) were loaded into each lane on the fractionator. For each lane a total of 150 µl from cleaned-up sample (see above) was rehydrated (as specified by Manufacturer's Protocol). IPG strips were also rehydrated with the Off-gel buffer prior to sample loading. Each sample was run in duplicate on 2 separate IEF lanes in the fractionator. The fractionator was set to run under the default Manufacturer's settings until the current was reduced to zero.

Samples were collected from each well and transferred into separate microcentrifuge tubes. The Bradford assay (see above) was performed on each fraction. Variation in protein concentrations between the duplicates remained within ±10%. Duplicates of each sample fraction were combined for further analysis. All Off-gel fractions were used for further processing.

Aliquots (25 µl) from each fraction were transferred into microcentrifuge tubes and contaminants removed as described above (see Sample clean-up). Duplicates were prepared for each sample. In the final step, the wash buffer was carefully decanted without disturbing the pellet. Pellets were allowed to further dry at 37.5°C in a heat block for 10 minutes to fully evaporate any wash buffer residue.

#### Lithium dodecyl sulfate –polyacrylamide gel electrophoresis (LDS-PAGE)

The dried pellet was resuspended in 5 µl LDS sample buffer (Invitrogen, Carlsbad, CA, USA) [23], 2 µl reducing agent (Invitrogen, Carlsbad, CA, USA) and 13 µl deionized water. The mixture was placed in a 37.5°C heat block for 10 minutes. Pre-cast 4–12% NuPage Bis-Tris 10 well Mini Gels (Invitrogen, Carlsbad, CA, USA) were used with 2-(N-morpholino) ethanesulfonic acid (MES)-SDS running buffer (Invitrogen, Carlsbad, CA, USA) diluted 1∶20. Injured and control samples were loaded as duplicates for each fraction. One lane of each gel contained a molecular weight standard (Novex® Sharp pre-stained standard, Invitrogen, Carlsbad, CA, USA). Gels were run at 200V constant voltage for approximately 35 minutes.

#### Silver Staining

Following electrophoretic separation, proteins in gels were silver stained for 10–15 min (Silver Stain Plus Kit, Bio-Rad Laboratories, Hercules, CA, USA) according to the Manufacturer's Protocol.

#### Densitometric Gel Analysis

Silver stained gels were scanned on a flatbed scanner (Agfa Duoscan, Mortsel, Belgium) and analysed using a 1D gel analysis software, GeneTools V4.01.02 (Syngene, Synoptics Ltd, Cambridge, England). The number of bands visible in each lane was counted. Each lane was checked for consistency between duplicates and protein profiles from injured spinal cord samples were compared to those from controls, for each fraction. Any differences in band intensity, staining or molecular weight changes were noted. A ±0.5 relative change threshold compared to control (set as 1) was accepted to identify proteins that changed their expression. This threshold was set after evaluation of technical variability of the methods employed [24].

### Mass Spectrometry

#### Tryptic digestion

Protein bands of interest were individually and manually excised from gels and de-stained in 50 mM ammonium bicarbonate and 50% acetonitrile. Gel pieces were washed and dehydrated in 50 mM ammonium bicarbonate and acetonitrile in alternating wash steps until completely dehydrated. Dehydrated gel pieces were rehydrated in 0.5 µg trypsin (Promega corp., Madison, WI, USA) and 20 mM ammonium bicarbonate solution for in-gel digestion. Bands were incubated at 37°C overnight and sonicated (Health Sonics, Livermore CA, USA) for 10 minutes prior to analysis. Tryptic digests were analysed using two mass spectrometry methods (see below).

#### LC-MS/MS

Tryptic digests were analysed by liquid chromatography-mass spectrometry/mass spectrometry (LC-MS/MS) using an HCT ULTRA ion trap mass spectrometer (Bruker Daltonics, Bremen, Germany) and a 1200 series Capillary HPLC (Agilent Technologies, Santa Clara, CA, USA). A Zorbax 300SB reversed phase column was equilibrated with buffer A (5% acetonitrile, 0.1% formic acid) at a flow rate of 10 µl/minute. Elution of sample was done with a 30 minute gradient from 0% to 55% buffer B (90% acetonitrile 0.1% formic acid). Electrospray ionization of the samples (HCT Ultra ion trap) was done under capillary voltage of 4000V dry gas at 300°C, flow rate of 8 L/minute and nebulizer gas pressure at 1500 mbar. This was followed by MS/MS analysis of peptides which were automatically selected under autoMSn mode after 1 minute. Data obtained from LC-MS/MS were searched against the National Center for Biotechnology Information (NCBI) non-redundant and Swiss-Prot databases using the MASCOT search engine (version 2.1, Matrix Science Inc., London, UK) with all taxonomy selected.

#### MALDI-TOF/TOF

Tryptic digests were additionally analysed using a matrix assisted laser desorption ionization-time of flight/time of flight (MALDI TOF/TOF) mass spectrometer (4700 Proteomics Analyser, Applied Biosystems, Foster City, CA, USA). Digested samples were spotted with a matrix solution of 10 mg/ml α-cyano-4-hydroxycinnamic acid (Laser BioLabs, Sophia-Antipolis, France) in 50% acetonitrile 0.1% trifluoroacetic acid and then analysed by mass spectrometry within mass range of 800–3500 Da, focus mass of 1400 Da at 1500 shots per spectra. The software (4000 Series Explorer version 3.0) automatically selected the 15 most intense peptides as precursor masses for ensuing MS/MS analysis. MS/MS analysis was carried out in reflectron mode with a relative precursor mass window of 50 resolution and spectra were summed at 2500 shots/spectrum.

All peptides were compiled and searched against the NCBI non-redundant and Swiss-Prot databases using the MASCOT search engine (version 1.9, Matrix Science Inc., London, UK) with all taxonomy selected.

### Ontological annotations of mass spectrometry results

Proteins identified by mass spectrometry were annotated using ontological terms and the publicly available online Gene Ontology (GO) database, AmiGO version 1.7 Database release 2-10-2010 (http://amigo.geneontology.org) which standardizes the representation of genes and their products by controlling the vocabulary used in terms of location, biological processes and molecular function using the *Homo sapiens* database. As results obtained from mass spectrometry were of non-human origin, each protein had to be matched to the human set using online database Basic Local Alignment Search Tool (BLAST, http://blast.ncbi.nlm.nih.gov/Blast.cgi) to search against the protein database using the identified protein sequence as the query. Parameters for the BLAST search were non-redundant databases searched against all organisms and protein-protein BLAST algorithms (blastp). A BLAST search was first performed against all organisms to identify sequence similarity. Search scores, sequence coverage and E-values for each protein were noted for all matched proteins. Only proteins matched to a human set that had >99% sequence coverage and low E-value (<0.0001) were used for the gene ontology search.

### Western blotting

Western blotting was performed on Off-gel fractions containing protein bands identified by mass spectrometry (Fractions 1–3 for 14-3-3 protein and Fraction 8 for ubiquitin, see below) and on protein extracts of spinal cords collected separately from those used for proteomic analysis in order to increase the biological reproducibility of this study (Table 2). LDS-PAGE was performed on all samples (20 µl for Off-gel fractions and 10 µl for homogenates) using 4–12% Bis-Tris gels (Invitrogen, Carlsbad, CA, USA) run at constant 200 V for 35 minutes in MES-SDS running buffer (Invitrogen, Carlsbad, CA, USA). Proteins were transferred from gels onto polyvinylidene fluoride (PVDF) membranes using an iBlot (Invitrogen, Carlsbad, CA, USA). Extracts from rat spinal cords were used as positive controls [25]–[27] and to confirm cross-reactivity of antibodies with *Monodelphis* tissue.

Following dry transfer, membranes were blocked in a solution of Tris-buffered saline (TBS, 0.1 M Tris, 1.5 M NaCl, with 0.1% Tween-20) with 50% low fat soy milk overnight at 4°C. PVDF membranes were incubated with primary antibodies diluted in blocking solution for 2 hours (for list of antibodies used and their specifications see Table 3), followed by incubations in secondary (swine anti-rabbit 1∶400, DAKO, Glostrup, Denmark) and tertiary (rabbit PAP 1∶400, Sigma-Aldrich, St Louis, MO, USA) antibodies for 2 hours each. Membranes were washed in TBS-Tween buffer between each antibody until the wash solution was clear. All incubations were done at room temperature (20°C). Bound antibodies were detected using diaminobenzidine (Sigma-Aldrich, St Louis, MO, USA). Membranes were washed in distilled water to stop the reaction and dried overnight. Images of membranes were taken using a Canon IXUS 80 s digital camera at 8 megapixels settings, superfine, digital macro-shot and white-balance corrected. Densitometry of each band was analysed using GeneTools V4.01.02 software (Syngene, Synoptics Ltd, Cambridge, England).

**Table 3:** Antibodies used in immunocytochemistry and Western blotting.

Primary Antibodies	Manufacturer	Immunocytochemistry (Dilution)	Western blotting (Dilution)	Characterization
**Anti-cow ubiquitin (rabbit)**	DAKO	1∶200	1∶200	Stains only expected band of ∼10 kDa on Western blot of *Monodelphis* spinal cord tissue which was confirmed by mass spectrometry (Figure 4)
**Anti-human pan-14-3-3 (rabbit)**	Biomol International	-	1∶500	Stains two expected bands ∼30 kDa of *Monodelphis* spinal cord tissue which was confirmed by mass spectrometry (Figure 4)
**Anti-N-terminus 14-3-3 epsilon (rabbit)**	Biomol International	-	1∶2000	Stains one expected band ∼30 kDa of *Monodelphis* spinal cord tissue which was confirmed by mass spectrometry (Figure 4C)

### Quantitative reverse transcription polymerase chain reaction (qRT-PCR)

Total RNA was extracted from spinal cords caudal to the lesion or corresponding regions of control animals using Qiagen RNeasy® mini kit (Valencia, California, USA) according to manufacturer's protocol. cDNA was reverse transcribed from equal amounts of total RNA using random primers and MultiScribe Reverse Transcriptase (Applied Biosystems, Foster City, California, USA). qRT-PCR was performed on these cDNA samples using SYBR Green chemistry with Taqman DNA polymerase and gene specific PCR primers (Table 4) based on the published genome sequence for *Monodelphis domestica* (MonDom5) available at Ensemble (http://www.ensembl.org/Monodelphis_domestica). The real time reactions were run on an Applied Biosystems 7300 Real-Time PCR System (Foster City, California, USA). All primer pairs were tested for specificity (by dissociation curves) and the concentration of cDNA was optimized for each primer pair such that the efficiency of the PCR reaction approached 100%. This was determined by running standard curves in which the slope should equal -3.3. All samples were run together in triplicate. Threshold cycle (Ct) values obtained for triplicates were averaged and normalized to a housekeeping gene (cyclophillin) for each RNA sample to obtain ΔCt values (ΔCt_gene of interest_ = Ct_gene of interest_ − Ct_cyclophilin_). Analyses were performed by the 2^−ΔΔCt^ method. After normalization to the housekeeping gene, ΔΔCt for the gene of interest was calculated by normalizing to the average value for the control samples (ΔΔCt = ΔCt_gene of interest (injured)_ − ΔCt_gene of interest (control)_). The change in experimental value (SCI) was calculated as a fold-increase or decrease relative to control (set equal to one) by calculating 2^−ΔΔCt^. All samples were run separately (not pooled) and the mean and standard error of the mean for each group was calculated (n = 6).

**Table 4:** Sequences of forward and reverse primers used in Quantitative RT-PCR.

Gene name	Forward primer	Reverse primer
**Cyclophilin**	5′-TCCAAAGGCAGCAGAAAACT	5′-AAAACTGGGAGCCATTTGTG
**Ubiquitin**	5′-GGTGGTGCCAAGAAGAGAAA	5′-ATAAAGACCCCAGCACCACA
**α-enolase**	5′-ATCTTTGACTCTCGCGGAAA	5′-CAACCGCTTTTGAGACACCT
**FABP 3**	5′-CTGGAAGCTGATTGACAGCA	5′-ACCCCCAACTTGAAGGAGAT
**FABP 7**	5′-AATGGTGGAGGCTTTCTGTG	5′-CTGGGTCCTGATCACCACTT
**Cofilin**	5′-ATCTTTGACTCTCGCGGAAA	5′-CAACCGCTTTTGAGACACCT
**14-3-3 ε**	5′-TGAAAGGGGACTACCACAGG	5′-CACGGTCAGGGGAATTAAGA
**14-3-3 γ**	5′-GAGCAACTGGTGCAGAAAGC	5′-TCCCACCACGTTCTTGTAGG

All primers used for qRT-PCR was designed by Jessie S. Truettner^3^.

### Statistics

Statistical evaluations were only performed on the qRT-PCR data using the Mann-Whitney U test (un-paired, two-tailed, non-parametric test) with Graphpad InStat (Ver. 3.0, San Diego, USA) using individual ΔCt values obtained from control and injured samples. P-values<0.05 were considered significant.

### Immunocytochemistry

Whole spinal cords (P8–P35) were removed whilst still within intact vertebral columns and immerse-fixed in Bouin's fixative overnight in room temperature followed by washes in 70% ethanol until clear, then embedded in paraffin wax and 5 µm transverse sections were cut on a Leica Microtome (Leica Microsysteme Vertrieb Gmbh, Wetzlar, Germany) and placed on silanised glass slides. Haematoxylin and Eosin (H&E) staining was done on every tenth slide for general morphology. Selected slides (every 10^th^ slide from each cord) were warmed at 60°C for 30 minutes to melt the paraffin wax, followed by dewaxing in two histolene chambers for 5 minutes each. Sections were rehydrated in graded alcohols (100%, 95% and 70%) for 5 minutes each and briefly washed in phosphate buffered saline (pH 7.3) with 0.2% Tween-20 (PBS/Tween20, Sigma-Aldrich, St Louis, MO, USA). Peroxidase and Protein Blockers (DAKO, Glostrup, Denmark) were used to block endogenous peroxidase activity and non-specific binding for 1 hour each, in moist chambers at room temperature. Primary and secondary antibody dilutions were made in PBS (pH 7.3) with 2% fish gelatin (Sigma-Aldrich, St Louis, MO, USA). For antibodies and their dilutions see Table 3. Sections were incubated overnight with the primary antibody at 4°C in moist chambers. This was followed by three 5 minute washes in PBS/Tween20. Sections were incubated with secondary (swine anti-rabbit, DAKO, Glostrup, Denmark) and tertiary (rabbit PAP, Sigma-Aldrich, St Louis, MO, USA) antibodies, both diluted 1∶200, for 2 hours each (Table 3). Staining was visualized using DAB+ Kit (DAKO, Glostrup, Denmark). The reaction was allowed to develop for approximately 5 minutes or until dark staining was observed and halted with a 10 minute wash in running distilled water. Sections were dehydrated in graded alcohols and histolene (3×5 minutes) and mounted with Ultramount #4 mounting medium (Fronine, Riverstone, NSW, Australia). Negative control sections were obtained by omitting the primary antibody and these always appeared blank. Positive control sections were of rat spinal cord tissue obtained from previous studies [28] and were always positive. Rat proteins are known to recognise the antibodies used [25]–[27].

## Results

**Figure 1:**
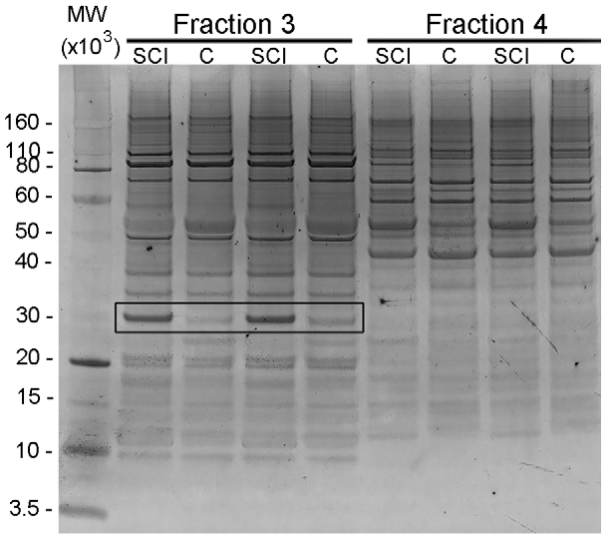
Silver stained gel of extracts from P28+1d SCI and P29 control (C) spinal cords. Shown are examples from two fractions, Fractions 3 and 4 (pH 4.16–4.74 and pH 4.74–5.32, respectively). Left lane: molecular weight marker (MW×10^3^). Next four lanes were duplicate samples from Fraction 3, followed by four lanes that were duplicate samples from Fraction 4. Black box indicates an example of a protein differentially expressed between control and spinal injury samples. This protein was later identified as 14-3-3 gamma.

In total, four injury groups (P7+1d, P7+7d, P28+1d and P28+7D) and four control groups (P8, P14, P29 and P35) of animals were studied. To obtain enough tissue for proteomic analyses, cords from several pups had to be pooled (see Table 2). In all age groups (except the youngest) 50–80 mg of pooled tissue was collected from pups from several litters to minimize biological variability. In the P7/P8 age group only 30 mg of tissue was collected; that amount was obtained from 18 individual pups from 5 separate litters (Table 2). Individual proteins were visualized using silver staining and were analysed by densitometry (Figure 1).

Each pooled sample was run in duplicate. Intensities of separated protein bands were compared to age-matched control samples and proteins that showed ±0.5 relative changes were analysed by mass spectrometry. Table 5 summarizes the proteins that were affected by spinal injury, the total number of proteins analysed by mass spectrometry and number of proteins that were successfully identified.

**Table 5:** Number of identified proteins that were differentially expressed identified after spinal cord injury (SCI) at P7 or P28 compared to age-matched controls 1 day (+1d) or 7 days (+7d) post injury.

Age group	P7+1d vs P8		P7+7d vs P14		P28+1d vs P29		P28+7d vs P35	
	Up-regulated	Down-regulated	Up-regulated	Down-regulated	Up-regulated	Down-regulated	Up-regulated	Down-regulated
**Number of differences**	23	55	14	16	65	46	36	7
**Total differences**	**78**		**30**		**111**		**43**	
**Bands analysed by mass spec**	23	55	0*	0*	55	44	36	7
**Total bands analysed by mass spec**	**78**		**0** *		**119**		**43**	
**Number of proteins identified**	11	24	7*	9*	45	39	18	4
**Total number of proteins identified**	**35**		**16** *		**84**		**22**	

*Identity of proteins obtained from mass spectrometry results from other age groups.

### Analysis of proteins in the spinal cord segment caudal to the site of transection

For pI-based separation using the Off-gel Fractionator [29], a 12-well set-up was used to separate samples within pH3–10 (resolution of 0.6 pH unit for each well, each well is termed a fraction) followed by gel electrophoresis for molecular weight separation prior to mass spectrometry. Six gels were run for each age and paired injured and control cord samples were run in duplicate in one gel (Figure 1).

A total of 24 gels were run and age-dependant differences, obtained from densitometry scans of protein bands, were identified. A list of full protein names and their abbreviations is provided in Table S1.The results are summarized in Tables 5 and 6 and are listed in Tables S2, S3, S4, S5.

**Table 6:** Proteins that were up-regulated following spinal injury, according to their biological functions.

Category	P7+1d	P7+7d	P28+1d	P28+7d
**Cell Death**	CFL1	GRP78	1433**ε**, CFL1, PKM2, Ub	1433ε/ζ, Alb, CFL1, ALDOC, GRP78, PKM2, Ub, VDAC1
**Nervous System Development**	-	GRP78	1433ε, CRMP2A	INA, GRP78, CRMP2A, DPYSL3, 1433ε, FABP3
**Cell Signaling**	CFL1	GRP78	1433ε, CFL1, Ub	1433ε, ACTα, CFL1, GRP78, PDH1, Ub
**Metabolism**	-	GRP78, TPI1	CRMP2A, PKM2	TUBα,TPI, PKM2, MDH1, LDHB, GRP78, ALDOC, CRMP2A, DPYSL3, ATP5B
**Protein degradation**	-	GRP78	-	TUBα, Ub, GRP78
**Secretory Function**	-	-	ANXA2, CRMP2A	1433ζ, CRMP2A
**Structural Organization**	CFL1, TPM1	GRP78	ANXA2, CFL1	TUBα, INA, GRP78, CFL1
**Protein Maturation**	CFL1, PPIAL, PPIB	GRP78	CFL1, PPIAL, PPIB, Ub	CCT2, CFL1, GRP78, PDH, Ub
**Inflammatory/injury response**	TPM1	GRP78	ANXA2	ATP5B, ALDOC, GRP78, LDHB, MDH
**Regulatory**	HNRNPAB, GTFII-I	1433**γ**, GRP78, HBZ	1433ε, CRMP2A, HBZ, Ub	Ub, PDH, HSP90β, GRP78, GTFII-I, CRMP2A, DPYSL3, ATP5B, ALB, 1433ε/ζ
**Protein transport**	CFL1, HNRNPAB, TPM1	HBZ, 1433γ	1433ε, CRMP2A, HBZ, VDAC1	1433ε/ζ, ALB, CFL1, CRMP2A, TUBα, VDAC1

Proteins were categorized according to their biological function and their response to spinal injury at P7 or P28. For individual age-groups refer to Tables S2, S3, S4, S5. – no proteins detected in this category.

In the comparison of P7+1d against P8, 23 proteins were up-regulated and 55 proteins were down-regulated after injury, while in the comparison of P7+7d against P14, 14 proteins were up-regulated and 16 proteins were down-regulated. In the comparison of P28+1d against P29, 65 proteins were up-regulated and 46 proteins were down-regulated and in the comparison of P28+7d against P35, 36 proteins were up-regulated and 7 proteins were down-regulated (Table 5).

In total, 240 protein bands were analysed by mass spectrometry resulting in 157 identity hits from which 56 unique proteins were identified. For each protein, its name, accession number, fraction it was identified from, theoretical molecular mass and sequence coverage identified by peptide mass fingerprinting are listed in Table S1.

### Ontological annotations of differentially regulated proteins following SCI

**Figure 2:**
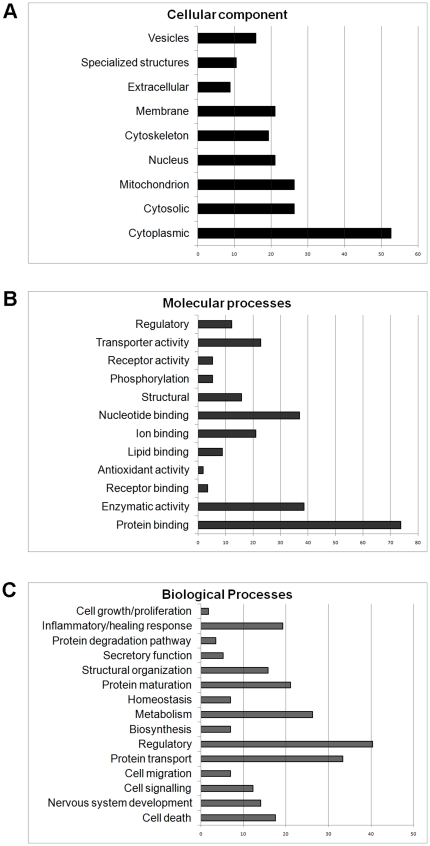
Ontological analyses of proteins identified by mass spectrometry that changed in response to spinal cord injury. Bar charts summarize the distribution of proteins that changed in response to spinal cord injury at all ages according to ontological categories (y-axis) and number of proteins (x-axis) in each category in terms of (A) cellular localization, (B) molecular function and (C) biological processes.

The 56 unique proteins identified following proteomic analysis and mass spectrometry had well-matched candidates within the eutherian mammal/*Monodelphis domestica* protein database and were classified according to their localization, molecular and biological functions based on information obtained from AmiGo (Version 1.7, http://amigo.geneontology.org) using the *Homo sapiens* database. Most of the proteins identified were predicted to be localized in the cytoplasm (>50%) and most were capable of molecular binding (>70%). Identified proteins were classified into 15 groups of function of which the main ones were regulation, transport, metabolism, protein maturation, inflammation, structure and cellular signalling (Table 6 and 7, Figure 2).

**Table 7:** Proteins that were down-regulated following spinal injury, according to their biological functions.

Category	P7+1d	P7+7d	P28+1d	P28+7d
**Cell Death**	1433ζ, HSP60, Ub, VDAC1	Alb, Ub, VDAC1	1433ζ	-
**Nervous System Development**	CRMP2A, DPYSL3, FABP7, INA, PFN1	FABP7	PFN1, TPM3	-
**Cell Signaling**	ACTα, PDH1, Ub, HSP60	Ub	ACTα	-
**Metabolism**	ATP5A1, ENO1, CRMP2A, DPYSL3, LDHB, MDH1, TPI1, TUBα	TUBα	ATP5A1, LDHB	GAPDH
**Protein degradation**	TUBα, Ub	TUBα	-	-
**Secretory Function**	1433ζ, CRMP2A	-	1433ζ	-
**Structural Organization**	DSTN, INA, PFN1, TUBα	TUBα	PFN1	-
**Protein Maturation**	CCT2, EEF1, HSP10/60, PDH, Ub	Ub	EEF1	-
**Inflammatory/injury response**	HSP10/60, LDHB, MDH1	-	LDHB	GAPDH
**Regulatory**	1433ζ, ENO1, ATP5A1, CRMP2A, DPYSL3, FABP7, GDI1, HSP60, INA1, PFN1, PDH, Ub	ALB, GTFII-I, Ub	1433ζ, ATP5A1, PFN1	HNRNPAB, HBZ
**Protein transport**	1433ζ, ATP5A1, CRMP2A, GDI1, HBA1, HBG2, TUBα, VDAC1	TUBα, Alb, VDAC1	1433ζ, ATP5A1, TPM3	HBA1, HBG2, HBE, HNRNPAB, HBZ

Proteins were categorized according to their biological function and their response to spinal injury at P7 or P28. For individual age-groups refer to Tables S2, S3, S4, S5. – no proteins detected in this category.

### Age related differential protein expression in response to spinal injury in the cord segment caudal to the transection

The results analysed by biological function (Figure 2 and Table 6 and 7) showed that after injury at P7 more proteins associated with CNS development, cell signalling, protein transport, metabolism, structural organization, protein maintenance, cell death and inflammation were decreased, but more were increased or modified after injury at P28 (Table 6 and 7). At P7, one day after injury, more proteins were up-regulated than were down-regulated. Conversely, more proteins were down-regulated one day after spinal injury at P28 than were up-regulated. Proteins that were down-regulated in the P7+1d group included 14-3-3 zeta, heat shock protein 60 (HSP60), ubiquitin, voltage dependent anion channel 1 (VDAC1), collapsin response mediator protein 2A (CRMP2A), dihydropyrimidinase like 3 (DPYSL3), adenosine triphosphate-5alpha-1 (ATP5A1) and heat shock protein 10 (HSP10). In contrast, proteins such as cofilin-1, tropomyosin, heterogenous nuclear ribonucleoprotein A2/B1 (HNRNPAB) and peptidylprolyl isomerise A-like (PPIAL) and B (PPIB) were up-regulated. At P7+7d, compared to P7+1d there was a decrease in the total number of proteins affected. Some of the proteins that were up-regulated at P7+7d included glucose regulated protein 78 (GRP78), tropomyosin and VDAC1. Some of the proteins that were down-regulated at P7+7d included ubiquitin, brain type fatty acid binding protein (FABP7) and heart type fatty acid binding protein (FABP3). In the P28+1d group, proteins such as annexin A2 (ANXA2), cofilin-1, PPIAL, PPIB, ubiquitin, CRMP2A and 14-3-3 epsilon were up-regulated. In contrast, proteins such as profilin-1, 14-3-3 zeta, ATP5A1, tropomyosin and lactate dehydrogenase (LDH) were down-regulated. At P28+7d, proteins such as 14-3-3 epsilon, 14-3-3 zeta, cofilin-1, fructose bisphosphate aldolase C (ALDOC), GRP78, VDAC1, FABP3, malate dehydrogenase 2, nicotinamide adenine dinucleotide (MDH1), DPYSL3, CRAMP2A and ubiquitin were up-regulated. Proteins such as HNRNPAB, several haemoglobin subtypes and glyceraldehyde-3-phosphate dehydrogenase (GAPDH) were down-regulated. For a complete list of proteins identified and their abbreviations refer to Table S1.

**Figure 3:**
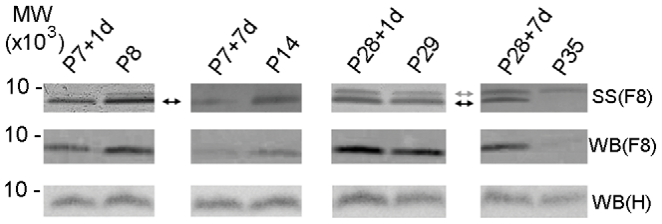
Silver staining and western blots of ubiquitin. Ubiquitin identified in silver stained (SS) gels and in western blots (WB) of Fraction 8 (F8) and in un-fractionated homogenate (H) of the cord segment caudal to the injury site. Ubiquitin was identified by mass spectrometry only in Fraction 8 (pH 7.06–7.64). Silver stained gels showed that in the P7-injured groups, a single band (black arrow) was detected. One day after injury (P7+1d) ubiquitin decreased compared to age-matched controls of P8 and the decrease was more pronounced seven days after injury (P7+7d). In the P28-injury groups, ubiquitin increased both 1 day (P28+1d) and especially 7 days (P28+7d) after injury compared to age-matched controls (P29 and P35 respectively). An additional band approximately 1 kDa higher (gray arrow) was also detected at these older ages. Western blots of Fraction 8 confirmed changes observed by silver staining. However, the antibody used was only able to detect the lower band and did not recognize the additional band observed in the P28-injury groups. Western blotting of the whole un-fractionated homogenate, (WB) H confirmed changes observed in silver stained gels although the differences were less pronounced. MW: Molecular weight marker (MW×10^3^).

**Figure 4:**
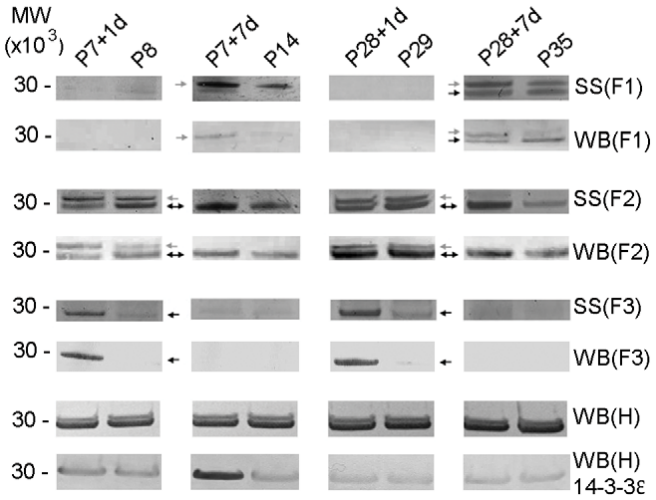
Silver staining and western blots of 14-3-3 protein family. Family of 14-3-3 proteins identified from silver stained gels (SS) and western blots (WB) of Fractions 1, 2 and 3 (F1, F2 and F3) and western blots of un-fractionated homogenate (WB) H of spinal cord segment caudal to the site of injury. Two subtypes, 14-3-3 epsilon and gamma were identified in silver stained gels and detected by mass spectrometry in F1, F2 and F3, pH 3.00–3.58, pH 3.58–4.16 and pH 4.16–4.76, respectively. 14-3-3 epsilon subtype was identified as the higher band (grey arrow) whilst 14-3-3 gamma subtype was identified as the lower band (black arrow). Results showed that 14-3-3 epsilon increased 7 days after injury at both ages (P7+7d and P28+7d) compared to age-matched controls (P14 and P35) in F1, while in F2 it increased 1 day after injury at P7 only (P7+1d). 14-3-3 gamma (lower band) was predominantly expressed in F2 at all ages but was additionally detectable in F1 at P35 and F3 at P8 and P29. In the P7-injured group, 14-3-3 gamma was initially down-regulated at 1 day after injury but was up-regulated 7 days after in F2. However in F3, it was up-regulated 1 day after injury but was not detectable at P14. In P28-injured group it showed differential expression in all fractions where it was down-regulated in F2 but was up-regulated in F3 one day after spinal injury. Seven days after injury, it was down-regulated in F1 but up-regulated in F2. Silver stained gels of F1-F3 were confirmed using pan-14-3-3 antibody and western blotting (WB). Western blotting of un-fractionated homogenates (H), which contain all 14-3-3 subtypes, with the pan-14-3-3 antibody showed no detectable overall changes in expression levels in the samples from spinal cords after injury compared to age-matched controls. Western blotting of un-fractionated homogenates with anti-14-3-3 epsilon showed that there was an increase in 14-3-3 epsilon expression at P7+1d and P7+7d compared to age-matched control. No changes were observed in P28-injured group compared to age-matched control. MW: Molecular weight marker (MW×10^3^).

Several proteins in each age group were identified in more than one fraction including some that showed changes after injury that varied depending on the fraction. One possible explanation for the presence of extracted proteins in multiple fractions is that these proteins may have been post-translationally modified resulting in either a change of isoelectric point or molecular weight. At P7+1d, such candidate proteins were GRP78, albumin, 14-3-3 gamma, GAPDH and several haemoglobin subtypes. An example at P7+7d was cofilin-1. In the P28+1d group, VDAC1, FABP3, 14-3-3 gamma, enolase-1, GAPDH, MDH1 and HSP10 were identified in more than one fraction suggesting a change in post-translational modifications. Lastly, at P28+7d, enolase-1 and 14-3-3 gamma also showed a possible difference in post-translational modifications after spinal injury. Figures 3 and 4 illustrate examples of two proteins identified by mass spectrometry (ubiquitin and 14-3-3) which showed age-related regulation of expression levels and potential modification in response to spinal injury (see below).

### Age-related changes in gene expression in response to spinal injury

**Figure 5:**
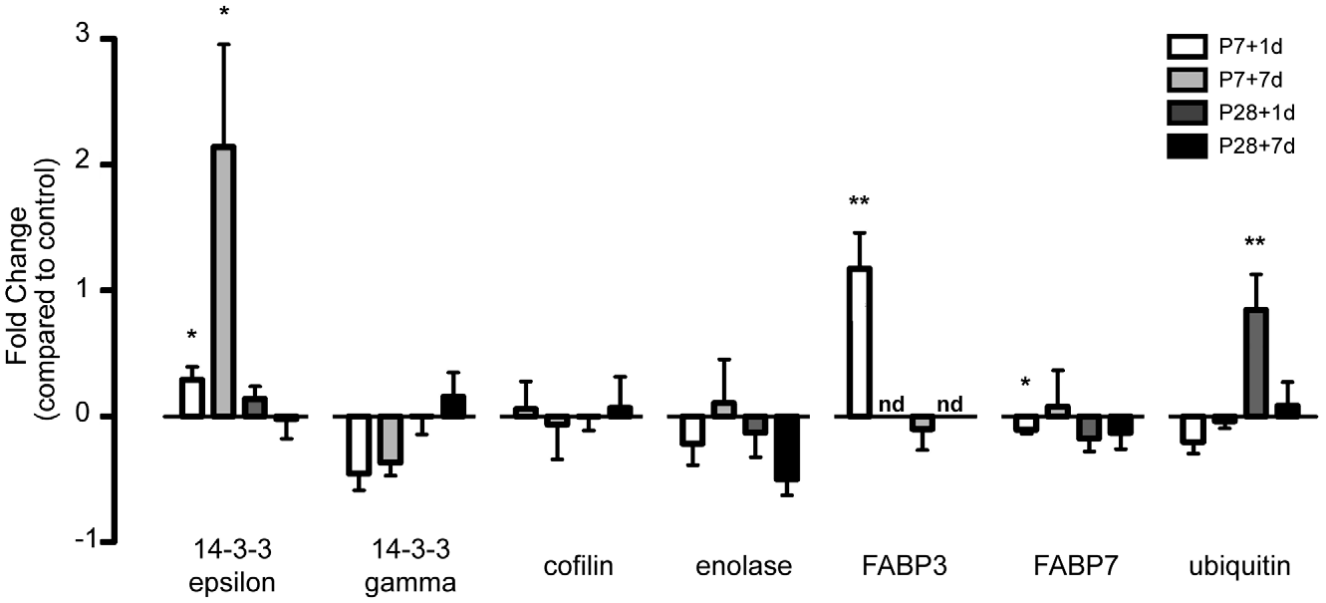
Expression values of genes validated by qRT-PCR at P7 and P28, 1 and 7 days after injury. Genes validated were 14-3-3 epsilon, 14-3-3 gamma, cofilin, enolase, ubiquitin and the fatty acid binding proteins 3 and 7 (FABP3, FABP7). Bars represent fold change normalized to age-matched controls with standard error of mean. Positive values represent up-regulation while negative values represent down-regulation. Mann-Whitney nonparametric statistical tests were used to show significance. Abbreviations: nd, (gene of interest) not detected; *, p>0.05–<0.10; **, p<0.05.

qRT-PCR was performed to examine levels of mRNA coding for seven of the proteins (14-3-3 epsilon, 14-3-3 gamma, cofilin, enolase-1, FABP3, FABP7 and ubiquitin) that showed differential age-related changes after spinal cord transection in the proteomic analysis (see above). Out of the 7 genes of interest, two genes: FABP3 at P7+1d and ubiqutin at P28+1d were significantly increased (p-value<0.05) with a fold change of 2.2±0.3 and 1.8±0.3 respectively when normalized to control (Figure 5, Table 8). Two other genes, 14-3-3 epsilon (at P7+1d and P7+7d) and FABP7 at P7+1d (Figure 5, Table 8) were approaching significance (p>0.05 to <0.10). 14-3-3 epsilon increased with a fold change of 1.3±0.1 at P7+1d and 3.1±0.8 at P7+7d compared to age-matched control. FABP7 decreased with a fold change of 0.9±0.03. All values are shown as 2^−ΔΔCt^±standard error of the mean (SEM). The other 3 genes (14-3-3 gamma, cofilin, enolase-1) did not show statistically significant changes at any age. mRNA for FABP3 at both P14 and P35 was not detectable. Results of fold changes for 7 proteins validated by qRT-PCR are shown in Figure 5 and Table 8.

**Table 8:** Statistical analysis of ΔCt values using un-paired, two-tailed Mann-Whitney U test.

Age	14-3-3epsilon	14-3-3gamma	cofilin	enolase	FABP3	FABP7	ubiquitin
**P7+1d**	0.0841*	0.3261	0.4675	0.4339	0.0031**	0.0878*	0.4231
**P7+7d**	0.0675*	0.2595	0.4906	0.7874	nd	0.5270	0.8943
**P28+1d**	0.1683	0.5903	0.3952	0.5935	0.5192	0.1994	0.0217**
**P28+7d**	0.9198	0.6656	0.8565	0.2286	nd	0.5924	0.4353

P-value<0.05 was considered statistically significant using Mann-Whitney U test. Tests were conducted on of ΔCt of injured groups compared to age-matched controls. Two genes, FABP3 at P7+1d and ubiquitin at P28+1d was statistically significant (**, p-value<0.05) in this study. Two other genes (14-3-3 epsilon at P7+1d and P7+7d and FABP7, both at P7+1d) were at p-value>0.05–p≤0.07 (*) and 3 genes did not reach significance at all ages studied (p-value>0.1). n.d. refers to FABP3 mRNA not being detected at ages P7+7d/14 and P28+7d/P35.

### Validation of proteomic results by western blotting

Results from the proteomic analysis were validated using western blotting for two proteins (ubiquitin and 14-3-3 protein family). Due to the numbers of cords required to pool to obtain enough material (see Table 1), all samples from P7-injured and control groups were run using a single pooled sample whilst all samples from P28-injured and control groups were run as duplicates using two pooled samples collected separately. For both proteins and for all animal groups, western blots were run on Off-gel fractions in which the proteins were identified and on un-fractionated homogenates of the spinal cord segment caudal to the injury, collected from different animal litters.

#### Ubiquitin

Western blotting for ubiquitin was performed only on Fraction 8 as this was the only fraction in which the protein was identified (Figure 3). This was followed by western blots of whole homogenates (un-fractionated samples). Results obtained from densitometry analysis of western blots of whole homogenates confirmed results obtained from silver stained gels and blots of Fraction 8 (Figure 3). Changes in response to injury were similar to those obtained from the proteomic analyses, showing a decrease in expression levels in the P7 group and an increase in the P28-injured group (Figure 3). However, only one band out of two identified in silver stained gels of Fraction 8 in P28 age group was recognized by the antibody (see Discussion).

#### 14-3-3 protein family

Mass spectrometry on two bands from silver stained gels identified three of seven subtypes that belong to the 14-3-3 family of proteins. Subtypes 14-3-3 gamma, epsilon and zeta were identified from the 2 bands located in 3 separate fractions (Fractions 1, 2 and 3) from silver stained gels (Figure 4). Western blotting using pan-14-3-3 rabbit antibody performed on all fractions confirmed the mass spectrometry results. Results showed that the upper band containing 14-3-3 epsilon was detectable only in Fractions 1 and 2. In Fraction 1, it was detectable only in P14 and P35 whilst in Fraction 2 it was detectable only at P8 and P29. The proteins in this band were clearly affected by spinal injury and were up-regulated at all times analysed after spinal transection.

The lower band containing 14-3-3 gamma was detected in three fractions, Fractions 1–3. The band was detectable at P35 in Fraction 1 where it was down-regulated 7 days after spinal injury at P28. It was detectable at all ages in Fraction 2 where it was down-regulated at P7+1d, up-regulated at P7+7d, not affected at P28+1d and up-regulated at P28+7d. In Fraction 3, it was only detectable at P8 and P29 and was up-regulated one day after spinal injury at P28. Western blots were also performed on whole homogenates with pan anti-14-3-3 proteins to confirm the total amount of the 14-3-3 protein. These blots did not show any clear differences between injury and control groups at any age, suggesting that overall, the total 14-3-3 protein levels may not have changed after injury, but individual subtypes did.

Western blots of 14-3-3 epsilon subtype on whole homogenates confirmed that there were changes in levels of expression of this subtype. 14-3-3 epsilon increased at P7+1d and at P7+7d compared to age-matched controls. In P28-injured groups, only a marginal change was observed in the injured animals compared to age-matched controls (Figure 4). Results obtained from western blots of 14-3-3 epsilon were similar to qRT-PCR data where gene expression was significantly increased at P7+1d and P7+7d but showed no changes at P28+1d and P28+7d compared to age-matched control (Figure 5).

### Immunocytochemistry

**Figure 6:**
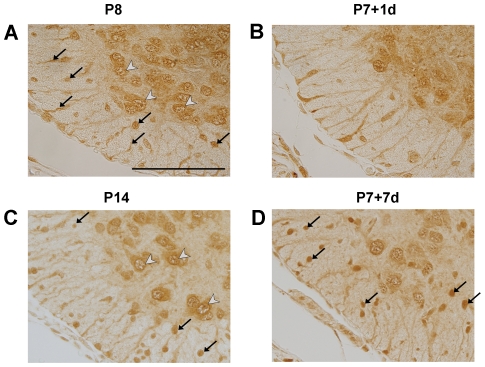
Immunocytochemical detection of ubiquitin in the spinal cord at P7+1d, P7+7d and their age-matched control groups. Ubiquitin staining at P8 (**A**), P7+1d (**B**), P14 (**C**) and P7+7d (**D**) of the ventral white and grey matter of the cord from section in the caudal to site of injury. At P8 (**A**) ubiquitin was predominantly localized in radial glia cells and their processes in the presumptive white matter (black arrows) and in nuclei of neuronal cell bodies located in the grey matter (white arrowheads). Following injury at P7 the general distribution of ubiquitin immunostaining was not different from controls (**B**). By P14 (**C**) immunoreactivity was prominent around nuclear membrane and was also visible in radial glia cells (arrows in **C**). Seven days after injury (**D**) the overall pattern of staining was similar to age-matched control (**C**) with the exception of more prominent immunostaining in small, rounded cells that are most likely developing glia (arrows in **D**). Identification of cell types was based on morphological appearance and in comparison with rat tissue where cellular markers are available (eg. Olig2 for oligodendrocytes) Scale bar: 100 µm.

**Figure 7:**
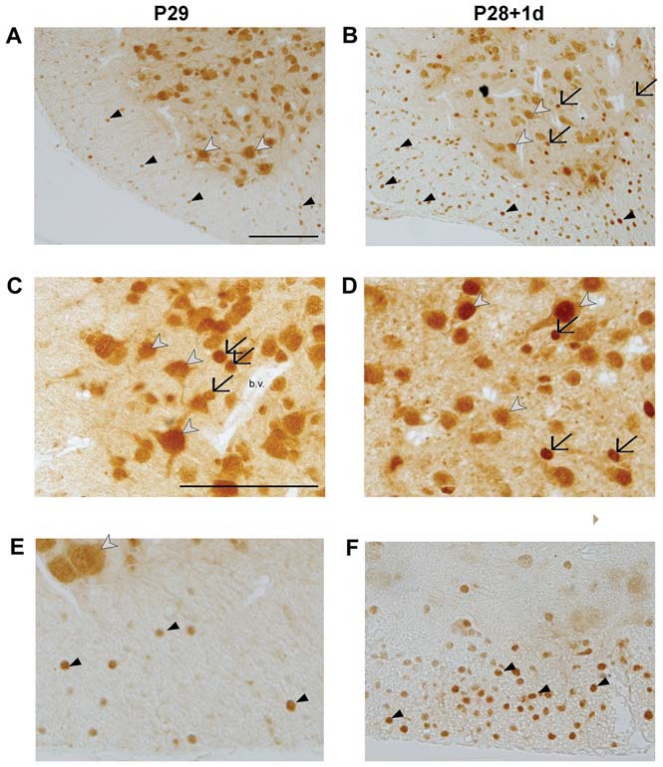
Immunocytochemical detection of ubiquitin in the spinal cord at P28+1d and P29. Immunocytochemical detection of ubiquitin at P29 (left panel) and P28+1d (right panel) of caudal section of the cord (**A and B**), grey matter (**C and D**) and white matter (**E and F**). In sections from both control and injured cords, ubiquitin was localized in nuclei of neurons (white arrowheads) and glial cells (black arrows) in the grey matter and oligodendrocytes in the white matter (black arrowheads). In addition, ubiquitin was detected in the cytoplasm of neurons. Identification of cell types was based on the morphology of the cell and comparison with rat tissue where cell-specific markers such as olig2 for oligodendrocytes and NeuN for neurons are available. Increased staining of oligodendrocytes (white matter) and glial cells (grey matter) was observed one day after injury as indicated by black arrows and arrowheads in (**B, D, F**). Similar pattern was visible 7 days after injury (not illustrated). Blood vessels (b.v. in **C**) were negative in all sections. All scale bars are 100 µm. Scale bar in **A** applies also to B. Scale bar in **C** applies to figures in **C**–**F**.

Immunological staining of paraffin-embedded sections through the 5 mm length of the spinal cord segment caudal to the injury was used to identify the cellular localization of ubiquitin and its response to SCI. Results are illustrated in Figures 6 and 7. In control spinal cords at all ages, ubiquitin is predominantly localized to neuronal cell bodies within grey matter. In younger spinal cord tissue (P7-injured groups and its age-matched controls), ubiquitin is additionally present within cell bodies and processes of radial glia (see arrows in Figure 6A). The radial glial immunoreactivity of ubiquitin is reduced at P14 and is no longer visible at P29 (Figure 7A) or P35 (not illustrated). At P14 ubiquitin immunoreactivity is also present within immature glial cell bodies both in white and grey matter (Figure 6C). By P29 and P35 ubiquitin can be identified in most neurons of grey matter, with its cellular localization being more nuclear (Figure 7A and C, white arrowheads). In addition, strongly stained glial cells in the white matter, with the morphology of oligodendrocytes, are also visible (black arrowheads in Figure 7E) as well as dispersed between grey matter neurons (black arrows in Figure 7D). Following SCI at P7, the pattern of immunostaining remained similar to control tissue at both one and seven days after injury (Figure 6B and D). At P7+7 days, compared to P7+1 day, more pronounced staining in glial cell bodies was present within the developing white matter (arrows in Figure 6D). This is in contrast to the observed changes following SCI at P28. At both times, one and seven days after injury, a noticeable increase in ubiquitin immunoreactivity in oligodendrocytes in grey and white matter could be observed (Figure 7A–F). There were also immunopositive glial cells within the grey matter (arrows in Figure 7C) in close apposition to negatively stained blood vessels (bv. in Figure 7C). A detailed study of the response of ubiquitin to SCI will be described elsewhere.

## Discussion

The aim of this study was to apply proteomic analysis to identify changes in protein expression in response to spinal cord transection at two developmental ages in the marsupial *Monodelphis domestica*: at P7, an age when functional recovery is associated with axon growth across the lesion [4], [7], or at P28, when axon growth does not occur [6], [8]. The two ages chosen in the present study also correspond to the time before myelination (P7) and the time when it is established (P28) [30]–[31]. The present study employed an electrophoretic technique, which allows rapid separation and visualization of many proteins within the proteome [32] including identification of possible post-translational modifications as suggested by changes in their pI and/or molecular mass. This was important in the identification of several proteins, where the possibility of post-translational modifications was represented by identification of the same protein in different fractions (e.g. cofilin and 14-3-3 protein, Table 6 and 7). The proteomic analysis of the spinal cord segment caudal to the injury site revealed 56 unique proteins that responded to spinal injury and two were validated using western blotting while seven were validated using qRT-PCR. In addition, ubiquitin was localized to specific cell types using immunocytochemistry. To date, proteomic analysis of *Monodelphis* spinal cord has not been performed, although two proteomic studies have been conducted on spinal cords from adult rats [33]–[34].

The pathophysiology of spinal cord injury at the lesion site has been well documented (reviewed in [35]; however, most studies have so far focused on understanding the neuronal ability to regenerate [36] and on studies of specific molecules within the CNS that are inhibitory to regeneration [37]). The aim of the present work was to identify changes in the proteome of the cord segment caudal to injury as a pioneering study towards a better understanding of the mechanisms of tissue remodeling following spinal trauma. Two time points after injury (one and seven days) were chosen in order to characterize major changes in the proteome' response as the injury progresses.

Independent studies using a marsupial cDNA microarray [17] and PCR-based subtractive hybridization [18] on spinal cord injured *Monodelphis* provide complementary information on gene expression for several proteins that have been affected by spinal injury. These proteins included several subtypes of the 14-3-3 protein family, VDAC1, TUBα, MDH1, ALDOC and CFL1. Additionally, although the gene for ubiquitin itself was not detected by the methods employed in these studies [17]–[18], several ubiquitin related proteins/enzymes were identified: ubiquitin-protein ligase E3 MDM2, ubiquitin-activating enzyme E1C and ubiquitin specific peptidase 8 and 12. However, a direct comparisons with the present study is difficult, because of differences in the experimental design (*in vitro* vs *in vivo*), ages used, type of injury (crush versus cut) and segment of cord sampled (whole cord vs caudal to injury)

### Proteomic and transcriptomic analyses of spinal cord response to injury

Results from proteomic analyses show that there was a difference in the protein response to injury at P7 compared to that at P28 (Table 5). In particular, more proteins changed their levels of expression one day than seven days after injury and more were down-regulated than up-regulated at P7 while more were up-regulated after injury at P28. Seven days after injury at P7 a similar number of proteins was up- and down-regulated while at P28+7d more proteins were up-regulated (Table 5). This result is of particular interest in view of previously published data demonstrating that one week following SCI at P7 (but not at P28) there is already a substantial re-growth of spinal cord tissue [7], [19]. However the interpretation of the results obtained in this study is complicated by two main factors. Although cords were pooled from several pups and from several litters in all age groups to provide a representative sample and eliminate biological variations within each group, proteomic analysis was only performed on one pooled sample run in duplicate. Therefore no statistical analysis was performed and a threshold for a significant difference was set at ±0.5 (compared to control which was set as 1) based on other published studies [24] and in consideration of the technical variability of the analytical method. However, using a relative change limits the sensitivity of the analysis because it excludes smaller variations that could potentially be biologically significant. To establish the biologically reproducibility of pooled samples in one experiment, cords from P35 control animals were collected from two separate groups of pups and analyzed simultaneously. Results showed that similar band patterns were obtained (not illustrated) indicating that differences seen between injured and control samples are likely to represent real biological differences.

The other complicating factor is that this study dealt with a developing CNS, thus some of the changes seen, particularly in samples collected seven days after injury, could be due to maturation processes that occur as part of normal development. For this reason all experimental samples were always paired with age-matched controls and were not directly compared between the two ages. Such a comparison would require a different experimental design and is currently being undertaken as a follow up- study.

Out of the seven genes tested by qRT-PCR (14-3-3 epsilon, 14-3-3 gamma, cofilin, enolase-1, FABP3, FABP7 and ubiquitin) mRNA for only two genes changed significantly (p<0.05, Figure 5): FABP 3 at P7+1d and ubiquitin at P28+1d. In the other age groups, although not significant, there was a trend for ubiquitin mRNA to decrease in the P7-injured group and increase in the P28-injured group. A similar change was observed in the proteomic analysis suggesting a direct relationship between the level of gene expression and the amount of protein present. Changes in two other genes approached significance (>0.05 to p<0.1), 14-3-3 epsilon at two ages, P7+1d and P7+7d and FABP7 at P7+1d.

Such a relationship is more difficult to establish for other proteins as they were identified in more than one fraction suggesting that some may have been post-translationally modified. Interestingly, the modifications were different after spinal injury. This indicates that other upstream regulatory proteins are being affected by the injury, leading to a difference in regulatory control over these downstream proteins. Additionally, FABP3 mRNA and its protein were both undetectable at two ages of the study (P7+7d and P28+7d and their corresponding age-matched controls) suggesting that this gene is expressed only at specific stages of development. However direct comparison between qRT-PCR data, which estimated mRNA extracted from the whole cord segment and proteomic data obtained from proteins separated into different fractions, is difficult.

### Functional classes of proteins that responded to spinal injury

A search conducted for each protein identified by mass spectrometry based on GO annotations showed that the identified proteins are involved in different biological processes affected by the injury in all age groups studied.

#### Proteins associated with nervous system development

The number of identified proteins involved in nervous system development, which showed a change in response to injury was similar in all injured groups (4–6 proteins) except at P7+7d when only one protein changed. This suggests that development of the spinal segment was affected by injury in all groups except at P7+7d, the time when cords are already undergoing a process of re-growth [19]. For example, the heart/brain fatty acid binding proteins (FABP3/7) have been shown to be critical in brain development [38]–[39]. In this study, FABP7 was down-regulated in P7 injured group but was not affected in the P28 injured group. FABP3 was identified from two fractions in which it was differentially regulated after injury. However, FABP3 protein was absent at P14 and P35, independently confirmed by qRT-PCR, which also showed the absence of its mRNA (see above). This suggests that the two FABP subtypes have different roles during development and in response to injury.

#### Proteins associated with cell death

Cell death can follow two main pathways, apoptosis or necrosis [40]. Whilst necrosis is predominantly an unwanted outcome from an injury [7], [40]–[41], apoptosis has been reported to play a dual role. Some evidence has indicated that apoptosis is beneficial to regeneration [42] whereas others have shown that prolonged apoptosis is detrimental due to the massive loss of neuronal tissue [43]. For example, HSP60 has been shown to be involved in regulating apoptosis and can function to either reduce or increase damage after injury [44]–[47]. In the present study HSP60 was down-regulated at P7+1d but was not changed in the other injured groups.

#### Proteins associated with cell signaling

The 14-3-3 protein family has been shown to be involved in numerous neuronal tissue signalling pathways [48]. One of the identified subtypes, 14-3-3 epsilon, has been reported to be involved in the demyelinating lesions in multiple sclerosis as part of the organization of the intermediate filaments in reactive astrocytes [49]. It is known that myelin in the opossums is not present at P7 but starts appearing around P28 [50]–[51]. Thus it is possible that this particular member of the family may be playing an important role in spinal injury. Its specific role needs further exploration in future studies.

Another protein that is known to play an important role in signalling is the glucose regulated protein 78 kDa (GRP78). This is a chaperone protein that specifically targets proteins that have been unfolded due to an insult [52]. However, as GRP78 is involved in various functions [53]–[55], its identification here only suggests that unfolded proteins may have been present after SCI.

#### Proteins associated with cell migration and structural organization

Several proteins that interact with the actin cytoskeleton have been identified and include cofilin and 14-3-3 epsilon. Cofilin is an actin depolymerization factor which disassembles the actin cytoskeleton [56]. Cofilin function is inhibited by LIM-kinase, an enzyme that is part of upstream proteins that regulate cytoskeleton dynamics [57]. 14-3-3 proteins have been shown to play an antagonistic role to LIM-kinases by activating cofilin through phosphorylation [58] and also binding to LIM-kinases to inhibit their function [59]. Cofilin has been shown to modulate growth cone guidance by altering the cytoskeleton network in neurons [60]. Both 14-3-3 epsilon and cofilin have been identified in this study in several fractions (cofilin in 5 fractions and 14-3-3 epsilon in 2 fractions). Other proteins identified that contribute to structural organization of the cytoskeleton include destrin, a member of the actin depolymerising factor/cofilin family [61] and profilin, shown to modulate the neuronal architecture using the actin cytoskeleton [62].

#### Proteins associated with protein transport

Several transport proteins including tubulin have been identified. Tubulin, if dysfunctional, has been shown to induce neuronal death in Parkinson's disease [63]. Other proteins in this group include cofilin, also part of the cytoskeleton network and several 14-3-3 proteins (gamma, epsilon and zeta), which have numerous regulatory roles that change in response to injury. Overall, the total number of transport-related proteins showing changes (up or down-regulation) was greater at P7+1d, P28+1d and P28+7d groups than at P7+7d.

#### Proteins associated with biosynthesis and homeostasis

ATP, synthesized by ATP synthase is critical for many cellular processes [64]–[65]. The present study identified two of the ATP synthase subtypes that interact to form the ATP synthase complex [66], ATP5A1 and ATP5B that were affected by injury. Both proteins were identified in several fractions and responded differently at each age which potentially could be due to a developmental difference in the spinal cord response to injury with respect to energy regulation. The results show that changes in regulation between the two subtypes did not correlate suggesting that there may be other factors affecting specific subtype expression levels. An additional observation was that ATP5B and several key enzymes involved in generating ATP were highly up-regulated at P28+7d and the opposite was true for the P7 injured animals.

#### Proteins associated with metabolism

Several enzymes directly involved in the glycolytic pathway have been identified. These are enolase, lactate dehydrogenase, malate dehydrogenase, triosphosphate isomerase, pyruvate kinase, aldolase and glyceraldehyde-3-phosphate dehydrogenase [67]–[69]. All of these enzymes were affected by injury, with age-related differential regulation: at P7+1d most were down-regulated, at P28+1d they were both up- and down-regulated in different fractions suggesting post-translational modifications and at P28+7d they were mostly up-regulated. However, at P7+7d, only triosephosphate isomerase was up-regulated after injury. This suggests that metabolism was decreased by injury in P7+1d cords and started to return to relatively normal levels seven days later, corresponding to the time when repair has commenced [19]. In P28 injured animals proteins associated with metabolism were still elevated seven days later, the time when no growth of axons occurs across the injury site.

#### Proteins associated with protein maturation and degradation

In the present study ten proteins involved in maturation of proteins were identified at P7+1d, a number that decreased to four by P7+7d. In the P28+1d and P28+7d groups eight such proteins were identified. Some key proteins involved in degradation pathways were annotated: HSP60, GRP78 and ubiquitin. HSP60 was down-regulated at P7+1d, but up-regulated at P7+7d. GRP78 was detected in more than one fraction and showed differential changes depending on the fraction. HSP60 and GRP78 showed no change at P28+1d, but GRP78 was up-regulated at P28+7d. In the P7-injured group ubiquitin expression was decreased which suggests that proteins were less actively targeted for degradation. Whilst current results are suggestive of an age-related difference in the degradation process following SCI, more in depth studies are required in order to understand the relationship between these proteins in response to injury at different ages.

#### Proteins associated with inflammation and known responses to injury

Inflammatory reactions have been shown to substantially contribute to the pathophysiology of spinal cord injury [70] and can be both beneficial as well as detrimental to recovery after an injury [71]. During the first week of life the opossum is incapable of initiating T-cell dependent immune responses due to the lack of circulating lymphocytes [72]–[73]. The acute phase response is also attenuated [74]. Both of these features of the immune system develop after two weeks of age. The proteomic results show that six proteins known to be involved in inflammatory responses in adults were affected at P7; TPM1, HSP10, HSP60, LDHB, MDH1 and GRP78. These proteins probably do not reflect an increased inflammatory response to injury, but may act as developmental factors involved in tissue remodelling. More of the proteins affected at this age were down-regulated, such as the heat shock proteins (HSP10 and HSP60), which are part of stress response mechanism and metabolic proteins (LDHB, MDH). The opposite was observed in P28 injured group where more of these proteins were up-regulated seven days after injury; ATP5B, ALDOC, GRP78, LDHB and MDH. Previously we have reported several inflammatory regulators that were affected by injury in the immature opossum spinal cord [19]. Most of these would not have been detected by the present analysis due to the differences in the sensitivity between protein detection and qRT-PCR.

#### Proteins associated with regulatory functions

GO analysis showed that 40% of the proteins identified in this study have regulatory functions (Figure 2) with the highest number (14) being affected by injury at P7+1d and lowest (6) at P7+7d. In contrast, in the P28 injured group the number of regulatory proteins affected increased between one and seven days after injury (7 to 13). This implies that regulation of various biological functions affected by injury plays an important role at the younger age, possibly resulting in protection of this tissue from further damage, whereas in the P28 injured group the response was slower and therefore less effective.

#### Ubiquitin and its response to spinal injury

Ubiquitin's role in protein degradation is widely known, where this protein acts as a tag to direct incorrectly folded or damaged proteins towards degradation. Thus, being a regulatory protein itself, it was interesting to note that ubiquitin underwent age-dependent post-translational modifications in response to spinal injury. Although still within one fraction on LDS-PAGE, ubiquitin was identified by mass spectrometry from two bands, the first of which was detectable by the antibody used on western blots. This band was identified in all age groups studied (Figure 3). The other band had a molecular mass heavier by 1 kDa and was only visible in silver stained gels and only in control animals aged P29 and P35. Further investigation of the mass spectrometry data showed that although the full sequence was identical between these two bands, their peptide fragments were different. This suggests that the cleavage sites recognized by tryptic enzymes used for digestion in mass spectrometry preparations were obscured, indicating the existence of age-dependent modifications to ubiquitin. Possible modifications include phosphorylation and glycosylation at this site, resulting in the observed increase of the molecular mass. Suggestions that ubiquitin changed both developmentally and in response to injury are supported by the results from the immunocytochemical studies. The cellular distribution of ubiquitin was different at different ages, especially between the two younger groups (P8 and P14) and the two older control groups (P28 and P35, Figures 5 and 6). Although immunocytochemistry is not a quantitative method, the results clearly show that the response to injury included differences in the protein levels particularly in the glial cells where they increased in the P28 injury group. This corresponds well with both qRT-PCR and western blot results where an increase in ubiquitin expression levels was seen. This response is in conjunction with developmental differences where ubiquitin cellular distribution changes from more cytoplasmic at P8 and P14 to more nuclear at older ages. Changes in cellular distribution of ubiquitin have previously been described in the developing rat brain (hippocampus and cortex) in association with neuronal maturation and differentiation [75]. In addition a similar cellular distribution to the opossum of ubiquitin in rat and human neurons was described [76], in particular on clastosomes, observed in the nucleus where intranuclear inclusion bodies enriched with ubiquitin and several other proteins associated with the ubiquitin-proteasome degradation pathway were localized [77]. It remains a possibility that age-dependent modifications would influence the ubiquitin response to injury and also offers a possibility of therapeutical interventions.

#### Potential drug treatments

A search of “DrugBank” (www.drugbank.ca) yields a number of interesting compounds available for targeting some of the proteins and their genes identified in this study that show developmentally regulated changes in response to injury. For example Bortezomib, which inhibits the mammalian 26S proteasome, has the potential to interfere with the ubiquitin pathway, which could be predicted to affect the up-regulation of ubiquitin identified in P28 injured animals. Another example is Muronomab, a monoclonal antibody, directed against the CD3 (T3) receptor on the surface of human T-cells (T-lymphocytes) which is one of five small molecules listed as interacting with 14-3-3 epsilon. Thus the next stage of the work utilizing this model of SCI could involve directly targeted functional studies.

### Conclusions and future directions

Results obtained in this study clearly indicate that proteomic analysis, western blotting, qRT-PCR and immunocytochemistry are approaches that yield complementary information to the study of age-related responses of the spinal cord to injury. Comparison of proteomic data with results obtained from qRT-PCR for the seven candidate genes indicated that there was a possibility that an injury could influence biological response at three levels of molecular function: (1) gene expression (mRNA) levels, (2) protein synthesis levels and (3) post-translational modifications of proteins. This indicates that other upstream regulatory genes or proteins not detectable by current methods are also affected by the injury. Combining these results with future studies of genomic regulation would provide a comprehensive overview of transcription, translation, modification and ultimately function of a protein's differential response to CNS injury during development. An important outcome from this study is the realisation that the protein and hence gene expression responses to injury in the immature spinal cord are complex. A similarly widespread gene expression response to injury (contusion) in adult rat spinal cord has already been reported [78]. It is not possible to deduce from the present study which of the changes in the proteome following spinal cord injury are directly contributing to the growth of axons that occurs at P7 but not at P28. Nor is it clear whether the relevant changes relate to the extracellular substrate through which the axons grow or to changes in the cells involved. However, the comprehensive changes in the proteome identified following spinal cord injury suggest that development of clinically useful therapies to improve the outcome of spinal cord injury patients may require understanding the contributions and interactions of many different pathways that appear to respond to injury. This would allow targeting of some of the upstream regulatory genes or proteins. The particular contribution of developmental studies such as described in this paper may be to define more clearly the pathways that promote axon growth compared to those at later ages that inhibit it, since the latter appear so dominant in the adult. This in turn may lead to novel approaches to development of effective therapies for spinal cord injury employing directed changes in expression of key upstream genes.

## Supporting Information

Table S1 **List of identified proteins.** Table lists full names including abbreviations, fraction(s) proteins were identified from, molecular mass in Dalton (Da) and peptide sequence and sequence coverage (%) used to identify each protein using mass spectrometry.
(DOC)

Table S2 **Mass spectrometry results for protein bands that changed due to spinal cord injury at P7+1d compared to P8 control.** Proteins are listed in alphabetical order in each group. Proteins listed in Multiple responses column refer to proteins which were identified from more than one fraction and were either up-regulated or down-regulated.
(DOC)

Table S3 **Mass spectrometry results for protein bands that changes due to spinal cord injury at P7+7d compared to P14 control.** Proteins are listed in alphabetical order. Proteins listed in multiple fractions refer to proteins which were identified from more than one fraction and were either up-regulated, down-regulated or show no change in any one of the fractions.
(DOC)

Table S4 **Mass spectrometry results for protein bands that changes due to spinal cord injury at P28+1d compared to P29 control.** Proteins are listed in alphabetical order. Proteins listed in multiple fractions refer to proteins which were identified from more than one fraction and were either up-regulated, down-regulated or show no change in any one of the fractions.
(DOC)

Table S5 **Mass spectrometry results for protein bands that changes due to spinal cord injury at P28+7d compared to P35 control.** Proteins are listed in alphabetical order. Proteins listed in multiple fractions refer to proteins which were identified from more than one fraction and were either up-regulated, down-regulated or show no change in any one of the fractions.
(DOC)
