# Supplementary material for: Age-Dependent Changes in the Proteome Following Complete Spinal Cord Transection in a Postnatal South American Opossum (Monodelphis domestica)
Source: PLoS One. 2011 Nov 16;6(11):e27465. doi: 10.1371/journal.pone.0027465 (PMC3217969; doi:10.1371/journal.pone.0027465)
Supplement: Table S1 — List of identified proteins. Table lists full names including abbreviations, fraction(s) proteins were identified from, molecular mass in Dalton (Da) and peptide sequence and sequence coverage (%) used to identify each protein using mass spectrometry. (DOC) [file pone.0027465.s001.doc]

| **Protein name** | **Abbreviation** | **Accession number** | **Da** | **Fraction(s)** | **Mascot search score** | **Sequence coverage (%)** | **Total peptides matched** |
| --- | --- | --- | --- | --- | --- | --- | --- |
| **14-3-3 𝝴 (match to mouse protein)** | 1433𝝴 | NP_033562 | 29170 | 1,2,3 | 156 | 46 | 11 |
| **14-3-3 γ** | 1433γ | XP_001378951 | 28271 | 1,2,3 | 465 | 36 | 7 |
| **14-3-3 𝝵** | 1433𝝵 | XP_001380146 | 34634 | 1,2,3 | 477 | 35 | 10 |
| **Aconitase 2** | Aco2 | XP_001378711 | 87034 | 10 | 76 | 5 | 4 |
| **Actin (β) isoform 1** | ACTA1 | XP_001362898 | 41710 | 4,5 | 449 | 39 | 12 |
| **Actin (γ)** | ACTAG2 | XP_001370868 | 41766 | 4 | 448 | 36 | 11 |
| **Albumin** | ALB | XP_001364858 | 68048 | 3,4,5,6,7,8,9,11 | 436 | 24 | 16 |
| **α-enolase (2-phospho-D-glycerate hydrolase)** | ENO1 | XP_001362200 | 47061 | 6,7,8,10,12 | 237 | 27 | 12 |
| **Annexin A2** | ANXA2 | XP_001374196 | 46964 | 8 | 82 | 10 | 4 |
| **Adenosine Triphosphate synthase α-subunit** | ATP5A1 | XP_001364704 | 59709 | 8,9 | 296 | 23 | 12 |
| **Adenosine Triphosphate Synthase β-subunit (mitochondrial)** | ATP5B | XP_001364069 | 55874 | 1,2,4 | 489 | 45 | 16 |
| **Casein-α-1 match to Bovine protein** | CASEIN | NP_851372 | 24513 | 9 | 79 | 21 | 3 |
| **Chaperonin containing-t-complex**  **polypeptide 1, β subunit** | CCT2 | XP_001369473 | 72938 | 6 | 120 | 5 | 3 |
| **Cofilin-1 match to sheep protein** | CFL1 | NP_001009484 | 18507 | 8,9,10,11,12 | 174 | 22 | 3 |
| **Chaperonin 10** | HSP10 | XP_001379358 | 27222 | 11 | 80 | 13 | 3 |
| **Collapsin response mediator protein 2A** | CRMP2A | XP_001371325 | 73683 | 6 | 83 | 17 | 8 |
| **Destrin** | DSTN | XP_001374193 | 18462 | 9,10 | 131 | 24 | 4 |
| **Dihydropyrimidinase like 3** | DPYSL3 | XP_001378663 | 74174 | 6 | 69 | 4 | 2 |
| **Elongation factor 1** | EEF1A1 | XP_001365660.1 | 50152 | 8,10,12 | 63 | 4 | 2 |
| **Fatty acid binding protein (Brain type)** | FABP7 | XP_001369599 | 14925 | 5 | 83 | 33 | 4 |
| **Fatty acid binding protein (Heart Type)** | FABP3 | XP_001381724 | 14806 | 5,6 | 108 | 23 | 3 |
| **Fructose-bisphosphate aldolase C by homolgy** | ALDOC | XP_001368691 | 39344 | 7,8 | 439 | 54 | 13 |
| **Guanosine diphosphate dissociation inhibitor 1** | GDI1 | XP_001362742.1 | 50584 |  | 45 | 3 | 1 |
| **General transcription factor II I Isoform 4** | GTFII-I | NP_001074217 | 107921 | 1 | 46 | 21 | 14 |
| **Glial Fribrillary Acidic Protein** | GFAP | XP_001368268 | 49654 | 4 | 117 | 13 | 6 |
| **Glucose regulated protein 78** | GRP78 | XP_001365714 | 72408 | 2,3 | 194 | 19 | 9 |
| **Glyceraldehyde 3 phosphate dehydrogenase** | GAPDH | XP_001364734 | 35961 | 9,10,11,12 | 165 | 23 | 7 |
| **Hemoglobin-α** | HBA1 | NP_001028158 | 15316 | 10 | 165 | 54 | 7 |
| **Hemoglobin embryonic-β chain** | HBG2 | XP_001365409 | 15978 | 10 | 49 | 23 | 3 |
| **Hemoglobin-𝝴** | HBE | XP_001365336 | 16111 | 8,10,11,12 | 499 | 55 | 7 |
| **Heterogenous nuclear ribonucleoprotein A2/B1** | HNRNPAB | NP_872591.1 | 32440 | 10,11,12 | 96 | 48 | 12 |
| **HSP90** | HSP90 | XP_001367371 | 84485 | 2 | 45 | 1 | 1 |
| **HSP1β match to mouse protein** | HSP1 | NP_032328 | 83229 | 9,10 | 58 | 4 | 3 |
| **HSP60 (mitochondrial)** | HSP60 | XP_001370003 | 61099 | 4 | 48 | 12 | 4 |
| **HSP84β** | HSP84 | XP_001367493 | 66226 | 3 | 154 | 24 | 9 |
| **Internexin neuronal intermediate filament-α** | INA | XP_001369078 | 55274 | 6 | 77 | 6 | 3 |
| **Lactate dehydrogenase** | LDHB | NP_001028150 | 36548 | 5 | 99 | 14 | 4 |
| **Lactoglobulin match to bovine protein** | LGB | NP_776354 | 19870 | 9 | 66 | 21 | 3 |
| **Malate dehydrogenase 2 , Nicotinamide adenine dinucleotide (mitochondrial)** | MDH1 | XP_001366592 | 35602 | 9,10,11 | 204 | 29 | 9 |
| **Neurofilament L subunit** | Nefl | XP_001372723 | 77043 | 2 | 187 | 20 | 15 |
| **Peptidylprolyl isomerase A-like** | PPIAL | XP_00137979 | 17919 | 8,9 | 98 | 39 | 7 |
| **Peptidylprolyl isomerase B** | PPIB | XP_001366685 | 23758 | 12 | 65 | 4 | 1 |
| **Pol polyprotein** | HBZ | XP_001372225 | 31211 | 10 | 47 | 15 | 4 |
| **Profilin** | PFN1 | XP_001366082 | 14996 | 11 | 74 | 11 | 1 |
| **Pyruvate dehydrogenase**  **(PDH)** | PDH | XP_001368538 | 117405 | 8 | 67 | 1 | 1 |
| **Pyruvate kinase (muscle)** | PKM2 | XP_001371268 | 57926 | 7,8 | 297 | 26 | 13 |
| **Transketolase** | TKT | XP_001379400 | 67914 | 7 | 152 | 14 | 8 |
| **Triosephosphate isomerase** | TPI1 | XP_001370168 | 26732 | 8 | 50 | 11 | 2 |
| **Tropomyosin 2 (fibroblast rat isoform 2)** | TPM1 | XP_001365835 | 32676 | 8 | 74 | 33 | 10 |
| **Tropomyosin 3 gamma isoform 8** | TPM3 | XP_001365838 | 29013 | 3 | 66 | 35 | 9 |
| **Tubulin-α** | TUBα | XP_001363356 | 50120 | 1,2,3,4 | 594 | 44 | 15 |
| **Tubulin-β** | TUBβ | XP_001368231 | 49875 | 1,2,3 | 351 | 34 | 11 |
| **Ubiquitin** | Ub | XP_001375230 | 17953 | 8 | 55 | 31 | 5 |
| **Vacuolar adenosine triphosphate synthase subunit A** | vATP5A1 | XP_001362881 | 68259 | 4 | 111 | 22 | 11 |
| **Voltage dependent anion channel 3** | VDAC3 | XP_001373131 | 30603 | 11 | 128 | 13 | 3 |
| **Voltage dependant anion selective channel protein 1** | VDAC1 | XP_001365887 | 30698 | 9,10,11 | 343 | 34 | 7 |
