# Supplementary material for: Age-Dependent Changes in the Proteome Following Complete Spinal Cord Transection in a Postnatal South American Opossum (Monodelphis domestica)
Source: PLoS One. 2011 Nov 16;6(11):e27465. doi: 10.1371/journal.pone.0027465 (PMC3217969; doi:10.1371/journal.pone.0027465)
Supplement: Table S2 — Mass spectrometry results for protein bands that changed due to spinal cord injury at P7+1d compared to P8 control. Proteins are listed in alphabetical order in each group. Proteins listed in Multiple responses column refer to proteins which were identified from more than one fraction and were either up-regulated or down-regulated. (DOC) [file pone.0027465.s002.doc]

| **Up-regulated** | **Down-regulated** | **Down-regulated** | **Multiple responses** |
| --- | --- | --- | --- |
| Cofilin | 14-3-3𝝵 | Heat shock protein 1-β | 14-3-3γ |
| General transcription factor II Isoform 4 | Actin-β isoform 1 | Heat shock protein 60 (mitochondrial) | Albumin |
| Heterogenous nuclear ribonucleoprotein A2/B1 | α-enolase (2-phospho D glycerate hydrolase) | Internexin neuronal intermediate filament-α | Glucose regulated protein 78 |
| Peptidylprolyl isomerase A-like | ATP Synthase alpha subunit | Lactate dehydrogenase | Glyceraldehyde-3-phosphate dehydrogenase |
| Peptidylprolyl isomerase B | Casein-α1 | Lactoglobulin | Hemoglobin 𝝴 |
| Tropomyosin 2 (fibroblast rat isoform 2) | Chaperonin containing-t-complex polypeptide 1, beta subunit | Malate dehydrogenase 2 , NAD (mitochondrial) | Heat shock protein 90 |
|  | Collapsin response mediator protein 2A | Profilin | Pol polyprotein |
|  | Destrin | Transketolase |  |
|  | Dihydropyrimidinase like 3 | Triosephosphate isomerase |  |
|  | Elongation factor 1 | Tubulin-α |  |
|  | Fatty acid binding protein (Brain type) | Tubulin-β |  |
|  | GDP dissociation inhibitor 1 | Ubiquitin |  |
|  | Hemoglobin-α | Voltage dependent anion selective channel protein 1 |  |
|  | Hemoglobin embryonic-β chain | Voltage dependent anion channel 3 |  |
