# Supplementary material for: Age-Dependent Changes in the Proteome Following Complete Spinal Cord Transection in a Postnatal South American Opossum (Monodelphis domestica)
Source: PLoS One. 2011 Nov 16;6(11):e27465. doi: 10.1371/journal.pone.0027465 (PMC3217969; doi:10.1371/journal.pone.0027465)
Supplement: Table S3 — Mass spectrometry results for protein bands that changes due to spinal cord injury at P7+7d compared to P14 control. Proteins are listed in alphabetical order. Proteins listed in multiple fractions refer to proteins which were identified from more than one fraction and were either up-regulated, down-regulated or show no change in any one of the fractions. (DOC) [file pone.0027465.s003.doc]

| **Up-regulated** | **Down-regulated** | **Down-regulated** | **Multiple responses** |
| --- | --- | --- | --- |
| 14-3-3 γ | Albumin | Ubiquitin | Cofilin |
| Glucose regulated protein 78 | General Transcription Factor Isoform 4 | Voltage dependent anion channel 1 | Hemoglobin 𝝴 |
| Ig alpha-I chain C | Brain type fatty acid binding protein |  |  |
| Pol polyprotein | Tubulin-α |  |  |
| Triosephophate | Tubulin-β |  |  |
