# Supplementary material for: Age-Dependent Changes in the Proteome Following Complete Spinal Cord Transection in a Postnatal South American Opossum (Monodelphis domestica)
Source: PLoS One. 2011 Nov 16;6(11):e27465. doi: 10.1371/journal.pone.0027465 (PMC3217969; doi:10.1371/journal.pone.0027465)
Supplement: Table S4 — Mass spectrometry results for protein bands that changes due to spinal cord injury at P28+1d compared to P29 control. Proteins are listed in alphabetical order. Proteins listed in multiple fractions refer to proteins which were identified from more than one fraction and were either up-regulated, down-regulated or show no change in any one of the fractions. (DOC) [file pone.0027465.s004.doc]

| **Up-regulated** | **Down-regulated** | **Multiple responses** |
| --- | --- | --- |
| 14-3-3 𝝴 | 14-3-3𝝵 | 14-3-3- γ |
| Annexin A2 | Actin-β isoform 1 | Albumin |
| Cofilin-1 | Actin-γ | Α-enolase (2-phospho D glycerate hydrolase) |
| Collapsin response mediator protein 2A | ATP synthase-α-subunit | ATP Synthase-β-subunit (mitochondrial) |
| Glial Fribrillary Acidic Protein (GFAP) | Chaperonin 10 | Fatty acid binding protein (Heart Type) |
| Peptidylprolyl isomerase A-like | Elongation factor 1 | Glyceraldehyde 3 phosphate dehydrogenase |
| Peptidylprolyl isomerase B | Lactate dehydrogenase | Hemoglobin 𝝴 |
| Pol polyprotein | Neurofilament L subunit | Heterogenous nuclear ribonucleoprotein A2/B1 |
| Pyruvate kinase (muscle) | Profilin | Heat shock protein 1-β |
| Ubiquitin | Tropomyosin 3-γ isoform 8 | Malate dehydrogenase 2 , NAD (mitochondrial) |
| Voltage dependent anion channel 3 |  | Tubulin-α |
|  |  | Voltage dependant anion selective channel protein 1 |
