# Supplementary material for: Age-Dependent Changes in the Proteome Following Complete Spinal Cord Transection in a Postnatal South American Opossum (Monodelphis domestica)
Source: PLoS One. 2011 Nov 16;6(11):e27465. doi: 10.1371/journal.pone.0027465 (PMC3217969; doi:10.1371/journal.pone.0027465)
Supplement: Table S5 — Mass spectrometry results for protein bands that changes due to spinal cord injury at P28+7d compared to P35 control. Proteins are listed in alphabetical order. Proteins listed in multiple fractions refer to proteins which were identified from more than one fraction and were either up-regulated, down-regulated or show no change in any one of the fractions. (DOC) [file pone.0027465.s005.doc]

| **Up-regulated** | **Up-regulated** | **Down-regulated** | **Multiple responses** |
| --- | --- | --- | --- |
| 14-3-3 𝝴 | Ig alpha I chain C region | Glyceraldehyde 3 phosphate dehydrogenase | 14-3-3 γ |
| 14-3-3 𝝵 | Internexin neuronal intermediate filament α | Hemoglobin α | Α-enolase (2-phospho D glycerate hydrolase) |
| Actin-βisoform 1 | Lactate dehydrogenase | Hemoglobin embryonic β chain | Elongation factor 1 |
| Albumin | Malate dehydrogenase 2 , NAD (mitochondrial) | Hemoglobin 𝝴 | Fructose-bisphosphate aldolase C |
| ATP synthase subunit β (mitochondrial) | Neurofilament L subunit | Heterogenous nuclear ribonucleoprotein A2/B1 |  |
| Chaperonin containing-t-complex polypeptide 1, beta subunit | Pyruvate dehydrogenase | Pol polyprotein |  |
| Cofilin-1 | Pyruvate kinase |  |  |
| Collapsin response mediator protein 2A | Transketolase |  |  |
| Dihydropyrimidinase like 3 | Triosephosphate isomerase |  |  |
| General transcription factor II I Isoform 4 | Tubulin α/β |  |  |
| Glucose regulated protein 78 | Ubiquitin |  |  |
| Heat shock protein 90 | Voltage dependant anion selective channel protein 1 |  |  |
